# Lineage structure and penicillin-binding protein variability in clinical *Streptococcus pneumoniae* isolates from Southwest China exhibiting reduced susceptibility to penicillin

**DOI:** 10.3389/fpubh.2026.1844601

**Published:** 2026-08-28

**Authors:** Chenglin Miao, Ziyi Yan, Li Liu, Xin Huang, Yingying Li, Linghan Kuang, Xingxin Liu, Jiaji Ling, Jingjing Luo, Huilian Chen, Xiaocui Huang, Xinghua Zhu, Yali Cui, Yi Xie, Yongmei Jiang

**Affiliations:** 1 Department of Laboratory Medicine, West China Second University Hospital, Sichuan University, Chengdu, China; 2 Department of Laboratory Medicine, West China Second University Hospital (Tianfu), Sichuan University (Sichuan Provincial Children's Hospital), Meishan, China; 3 Key Laboratory of Birth Defects and Related Diseases of Women and Children (Sichuan University), Ministry of Education, Chengdu, China; 4 Department of Blood Transfusion, Beijing Anzhen Nanchong Hospital of Capital Medical University & Nanchong Central Hospital, Nanchong, China; 5 School of Medical Technology, Chengdu University of Traditional Chinese Medicine, Chengdu, China; 6 Department of Laboratory Medicine, Chengdu Hi-Tech Zone Hospital for Women and Children (Chengdu Hi-Tech Zone Hospital for Maternal and Child Healthcare), Chengdu, China; 7 Department of Laboratory Medicine, Meishan Women and Children’s Hospital, Meishan, China; 8 Department of Laboratory Medicine, Chengdu Jinjiang District Maternal and Child Healthcare Hospital, Chengdu, China; 9 Department of Laboratory Medicine, The First People’s Hospital of Longquanyi District, Chengdu, China; 10 Department of Laboratory Medicine, Clinical Laboratory Medicine Research Center, West China Hospital, Sichuan University (Sichuan Clinical Research Center for Laboratory Medicine), Chengdu, China

**Keywords:** antimicrobial resistance, China, global pneumococcal sequence cluster, GPSC, PBPs, penicillin, penicillin-binding proteins, *Streptococcus pneumoniae*

## Abstract

**Background:**

Reduced susceptibility to penicillin in *Streptococcus pneumoniae* is mediated primarily by alterations in penicillin-binding proteins (PBPs) and often coexists with multidrug resistance within successful lineages. The region-specific genomic characterization of clinically relevant pneumococci with reduced penicillin susceptibility in Southwest China remains limited.

**Methods:**

We performed whole-genome sequencing of 204 clinical *S. pneumoniae* isolates collected from five institutions in Southwest China (2018–2022) that met our operational screening definition of reduced susceptibility to penicillin (PEN MIC ≥0.12 μg/mL). Molecular serotypes, MLST types, and Global Pneumococcal Sequence Clusters (GPSCs) were assigned; virulence and antimicrobial resistance determinants were profiled; and a core genome phylogeny was reconstructed with international contextualization through the use of PubMLST genomes meeting the same MIC criterion. Amino acid variability in PBP1a/PBP2b/PBP2x was quantified using TIGR4 numbering, and highly variable noncatalytic residues located within 15 Å of catalytic motifs were prioritized via structure-guided screening.

**Results:**

The isolates showed a high burden of resistance to non-β-lactam antibiotics (erythromycin, 98.5%; tetracycline, 82.8%; trimethoprim–sulfamethoxazole, 64.7%), while fluoroquinolone susceptibility was largely preserved (≥97%), and vancomycin/linezolid resistance was not detected. Twenty-seven serotypes were identified, among which 19F (23.5%) and 19A (14.2%) were dominant, and the estimated PCV13 coverage was 69.6%. GPSC1 was the dominant lineage (36.8%), and the lineage composition among our isolates differed markedly from those in the PubMLST-USA and PubMLST-Thailand subsets. Virulence and resistance gene carriage differed markedly between GPSC1 and non-GPSC1 isolates, with enrichment of pilus operons, *mef(A)/msr(D)*, and *folA/folP* in GPSC1. PBP variations were clustered in transpeptidase domains and motif-adjacent regions while essential catalytic residues were conserved; with the structure-guided filter, 12, 11, and 11 motif-proximal noncatalytic candidate sites were prioritized in PBP1a, PBP2b, and PBP2x, respectively.

**Conclusion:**

Clinical *S. pneumoniae* isolates with reduced penicillin susceptibility collected in Southwest China demonstrated resistance and accessory gene profiles that were strongly structured by a GPSC-defined lineage background. Our site-resolved, structure-guided PBP analysis provides a regional PBP variability landscape and a compact set of recurrent motif-proximal candidate substitutions to support surveillance and downstream functional validation.

## 1 Introduction

*Streptococcus pneumoniae* is a major human pathogen that asymptomatically colonizes the nasopharynx but can invade to cause pneumonia, bacteremia/sepsis, and meningitis (1). In the Global Burden of Disease Study 2019, pneumococcus remained a prominent contributor to bacterial pathogen–associated mortality worldwide, underscoring its persistent clinical and public health relevance despite advances in prevention and care (2, 3). In young children, pneumococcal disease has historically accounted for a substantial proportion of global deaths from pneumonia and invasive disease, and its residual burden continues to be shaped by the heterogeneous serotype distribution and differential vaccine coverage across regions (4, 5).

β-Lactams remain central to the treatment of pneumococcal infections, although the preferred agent varies according to the clinical syndrome, disease severity, local resistance patterns, and antimicrobial susceptibility findings (6, 7). At the population level, antimicrobial resistance (AMR) is a leading determinant of adverse outcomes and healthcare burden, with pneumococcus among the pathogens contributing meaningfully to global AMR-attributable mortality (8). Importantly, the label “penicillin-nonsusceptible *S. pneumoniae* (PNSP)” is breakpoint-dependent and clinically contextual: the CLSI penicillin breakpoints differ for meningitis versus nonmeningitis syndromes and have been revised, making it essential to state an explicit operational definition in clinical genomic studies when cohorts enriched for elevated penicillin MICs are being assembled (9, 10). Mechanistically, reduced β-lactam susceptibility in pneumococci is driven primarily by alterations in penicillin-binding proteins (PBPs), which often arise through recombination and mosaic events at *pbp* loci, and these changes frequently cosegregate with other resistance determinants within successful lineages (11, 12).

Vaccination has reduced the disease burden attributable to vaccine-included serotypes, yet serotype replacement and the expansion of drug-resistant nonvaccine serotypes and lineages, including serotypes 24F and 11A, have increasingly shaped postvaccine epidemiology in multiple geographic regions (11–15). In Asia, surveillance studies have documented dynamic shifts in pneumococcal serotype distributions alongside persistently high rates of macrolide resistance and substantial β-lactam nonsusceptibility, with the emergence or persistence of drug-resistant serotypes becoming an ongoing concern (16, 17). In China specifically, pre-PCV and early-PCV era syntheses and hospital-based studies similarly indicate notable resistance burdens and geographic variations in serotype structures, highlighting the need for regionally grounded genomic characterizations focused on clinically consequential subpopulations (18, 19). Because the AMR and antigenic composition of pneumococci are strongly structured by the phylogenetic background, modern genomic surveillance increasingly relies on standardized lineage frameworks (e.g., Global Pneumococcal Sequencing Clusters, GPSCs) and large, curated genome libraries (e.g., PubMLST/BIGSdb resources) to enable cross-study comparability and robust international contextualization (20, 21).

In this study, we focus on clinical *S. pneumoniae* isolates exhibiting reduced susceptibility to penicillin collected in Southwest China using a prespecified MIC-based enrichment strategy referenced to the CLSI meningitis breakpoint; for study inclusion, reduced susceptibility was operationally defined as a penicillin MIC ≥0.12 μg/mL. We integrated these isolates with publicly available genomic data to place the regional findings in a broader global context. We apply whole-genome sequencing to define the serotype and GPSC composition, reconstruct phylogenetic relationships, and characterize the distribution of *pbp* variation together with virulence- and antibiotic resistance-related loci across lineages, thereby providing an integrated clinical–genomic portrait of pneumococcal isolates with elevated penicillin MICs from Southwest China and clarifying the relationship of this cohort to globally sampled populations.

## 2 Materials and methods

### 2.1 Isolates enrolled

We retrospectively collected clinical *S. pneumoniae* isolates from January 2018 to December 2022 at West China Second University Hospital, West China Hospital, Meishan Women and Children’s Hospital (Alliance Hospital of West China Second University Hospital), First People’s Hospital of Longquanyi District (West China Longquan Hospital) and Chengdu Jinjiang Hospital for Women’s and Children’s Health, a set of hospitals including one of China’s largest specialty hospitals for children and women, one of China’s largest general hospitals, and representative secondary and tertiary specialty or general hospitals. The clinical laboratories of these hospitals have been accredited by the College of American Pathologists (CAP) or the China National Accreditation Service for Conformity Assessment (CNAS) under the ISO 15189 accreditation standard or are under the supervision of the aforementioned external quality assessment laboratories.

In this study, clinical *S. pneumoniae* isolates exhibiting reduced susceptibility to penicillin were operationally defined as those with a penicillin minimum inhibitory concentration (MIC) ≥ 0.12 μg/mL, with the Clinical and Laboratory Standards Institute (CLSI) meningitis breakpoint used as the screening threshold (22). This cutoff was applied to enrich the cohort for isolates with elevated penicillin MICs for downstream phenotypic and genomic analyses. For isolates recovered from non-cerebrospinal fluid specimens, this definition was used for analytical stratification rather than to replace infection site-specific clinical susceptibility interpretations.

### 2.2 Isolate identification and antibiotic susceptibility testing

*Streptococcus pneumoniae* isolates were collected, cultured, and identified according to standard clinical procedures as previously described (23, 24). Blood, cerebrospinal fluid, and chest drainage samples were cultured using the BD BACTEC FX system (BD, MD, USA) and subcultured on Columbia agar with 5% sheep blood plates, whereas other samples were directly cultured on the same medium at 35 °C for 24–48 h in 5% CO_2_. Isolates were identified by MALDI-TOF MS (Vitek MS; bioMérieux), confirmed by whole-genome signature sequence alignment with PubMLST, and then stored in 25% sterile glycerol broth at −70 °C for subsequent analysis.

Antibiotic susceptibility was tested by the broth turbidity method using AST dishes (TDR STR-AST; Mindray, China) to evaluate susceptibility to penicillin, amoxicillin, cefotaxime, meropenem, erythromycin, tetracycline, trimethoprim–sulfamethoxazole, ofloxacin, levofloxacin, moxifloxacin, linezolid and vancomycin. Quality control was performed with the *S. pneumoniae* ATCC 49619 strain, and the testing procedures and interpretation of the results were conducted in accordance with the manufacturer’s instructions and the CLSI 2021 guidelines (22). The MIC₅₀ and MIC₉₀ were defined as the lowest tested antimicrobial concentrations encompassing at least 50 and 90% of the isolates, respectively, on the basis of the ordered twofold-dilution MIC distribution.

### 2.3 Genome sequencing and molecular typing

Genomic DNA was extracted using a QIAamp DNA Mini Kit (QIAGEN, Germany), and libraries were prepared with an NEBNext Ultra DNA Library Prep Kit for Illumina (NEB, USA). Whole-genome sequencing was performed on the Illumina NovaSeq PE150 platform with ~200× coverage (Beijing Novogene, China). Reads were assembled with SPAdes (v3.14.1) and annotated with Prokka (v1.14.5) as previously described (25), and the assemblies were deposited in GenBank (PRJNA914101, PRJNA915833, and PRJNA915821).

Molecular serotypes were determined from genome assemblies using SeroBA (v2.0.4) implemented in Pathogenwatch^1^ (26). Sequence types were assigned using the *S. pneumoniae* MLST scheme in the PubMLST database in conjunction with the mlst tool (v2.19.0) (21). Global Pneumococcal Sequence Cluster (GPSC) designations were obtained using the Pathogenwatch platform (see text footnote 1).

### 2.4 Phylogenetic analysis

We downloaded all records available in the PubMLST Pneumococcal Genome Library as of July 31, 2025. Genomes with available penicillin minimum inhibitory concentration information were screened, and the corresponding genome assemblies in FASTA format were retrieved for downstream analyses. All downloaded assemblies were reprocessed through our quality control pipeline and annotated with Prokka (v1.14.5) (25), and molecular serotypes were revalidated using SeroBA (v2.0.4) implemented in Pathogenwatch (see text footnote 1) (26).

Roary was used to identify the core genome and to generate a concatenated multiFASTA alignment of core genes present in more than 99% of the isolates (27). To avoid redundancy in the downstream phylogenetic inference, duplicate sites in the core gene alignment were removed using snp-sites (v2.3.3). The resulting alignment was subsequently used to infer maximum likelihood phylogenies with RAxML (v8.2.10) under the GTRGAMMA substitution model (28). The final trees were visualized and further annotated in iTOL (v6, https://itol.embl.de).

### 2.5 Virulence gene and antibiotic resistance-related gene screening

Virulence gene screening was conducted with ABRicate (v1.0.1) against the Virulence Factor Database (VFDB), covering genes associated with exotoxins, exoenzymes, immune modulation, nutritional and metabolic factors, adherence, and pilus-1 and pilus-2 (29). Hits meeting an identity cutoff of ≥80% were considered positive, and the corresponding isolates were classified as carriers of the respective virulence genes. Antimicrobial resistance gene profiling was also performed using ABRicate (v1.0.1) with multiple reference databases, including NCBI AMRFinderPlus, the Comprehensive Antibiotic Resistance Database (CARD), and ARG-ANNOT.

### 2.6 Penicillin-binding protein analysis

The protein sequences of PBP1a, PBP2b, and PBP2x were retrieved from the 204 clinical *S. pneumoniae* isolates. For each PBP, the amino acid sequences were aligned using MAFFT (v7.490) (30). The aligned sequence from the penicillin-susceptible reference strain TIGR4 was used as the positional reference for downstream analyses.

For quantification of site-specific variability, we calculated (i) the overall variation rate (proportion of isolates whose residue differed from that of TIGR4 at that position) and (ii) the substitution spectrum (frequency of each amino acid among the isolates). To avoid artifacts from rare indels, a position was retained only if the nongap support percentage was ≥ 99%. In addition to this unbiased site-by-site analysis, the complete variation profiles were reviewed at previously reported β-lactam resistance-associated PBP signature positions and regions. This literature-guided comparison was performed independently of the subsequent structure-guided candidate-site filter.

To prioritize substitutions likely to affect β-lactam binding while preserving essential enzymatic function, we performed a structure-guided screen for highly variable noncatalytic sites near the active site pocket. The following crystal structures were obtained from the Protein Data Bank and analyzed: PBP1a: 2C6W, PBP2b: 2WAF, and PBP2x: 5OIZ. TIGR4 reference sequences were mapped to each PDB chain by pairwise sequence alignment, generating a residue correspondence table restricted to residues present in the structure (with Cα coordinates available). Catalytic motifs [SXXK, SXN, and K(T/S)G] were defined on the mapped structures, and catalytic residues were excluded from candidate selection [S and K in SXXK; S and N in SXN; all residues in K(T/S)G; and the “X” positions in SXXK/SXN were retained]. For each mappable noncatalytic position, we computed the minimum Cα–Cα distance to any catalytic residue. The threshold criteria for defining candidate resistance-associated sites were defined as follows: a variation rate at or above the protein-specific 95th percentile among high-support, structurally mappable noncatalytic positions and a minimum Cα–Cα distance of ≤15 Å from a catalytic-motif residue.

As an exploratory genotype–phenotype extension, we evaluated the relationship between isolate-specific penicillin MIC and the full PBP variation dataset rather than restricting the analysis to selected residues. The analysis encompassed all high-support variable native TIGR4 positions, the aligned sequence profiles of each PBP and the three PBPs jointly, the per-isolate non-TIGR4 residue burden, the structure-guided candidate positions listed in Table 1, and insertion events occupying alignment columns that were gaps in TIGR4. To summarize recurrent motif-related combinations in an interpretable form, we additionally constructed a focused three-PBP profile from 12 literature-supported or cohort-recurrent positions highlighted by the variation analysis: PBP1a positions 371, 432, and 574–577; PBP2b positions 446 and 619; and PBP2x positions 338, 339, 394, and 546. This focused panel was used for descriptive presentation and did not restrict the alignment-wide analysis.

**Table 1:** Structure-guided candidate PBP substitutions in the 204 clinical isolates exhibiting reduced susceptibility to penicillin collected in Southwest China.

Protein	Site	RefAA	Variation rate	Minimum Cα–Cα distance (Å)
PBP1a	546	N	0.946078	9.901376
	577	F	0.946078	14.10486
	583	L	0.936275	4.768383
	585	A	0.936275	5.73339
	459	I	0.897059	12.67625
	611	L	0.867647	10.27499
	612	T	0.867647	10.29981
	606	L	0.867647	13.09766
	462	S	0.857843	10.08929
	432	P	0.848039	5.618353
	414	G	0.848039	9.627192
	397	E	0.848039	10.07165
PBP2b	446	T	0.975490	3.74885
	489	T	0.970588	14.34237
	438	Q	0.926471	7.957983
	455	L	0.916667	14.72637
	412	S	0.901961	9.603273
	426	T	0.838235	8.155183
	427	Q	0.818627	11.85399
	422	N	0.813725	6.838616
	480	S	0.789216	13.78216
	630	T	0.593137	9.52551
	597	G	0.401961	12.31277
PBP2x	567	D	0.985294	13.84415
	385	T	0.980392	10.94408
	576	S	0.955882	12.25673
	382	G	0.941176	13.16773
	389	S	0.931373	10.3695
	565	L	0.931373	13.73237
	605	N	0.931373	14.01843
	401	T	0.901961	6.471215
	384	R	0.901961	10.44033
	516	V	0.897059	8.606879
	358	V	0.892157	12.81966

Candidate sites were defined separately for each PBP as high-support (Effective N/204 ≥ 0.99), structurally mappable, noncatalytic positions with a variation rate at or above the protein-specific 95th percentile and a minimum Cα–Cα distance of ≤15 Å from any residue within the SXXK, SXN, or K(T/S)G motif. Catalytically essential residues were excluded from candidate selection [S and K in SXXK; S and N in SXN; and all residues in K(T/S)G]. TIGR4-to-structure residue mapping was performed using PBP1a structure 2C6W chain B, PBP2b structure 2WAF chain A, and PBP2x structure 5OIZ chain A; the third motif in PBP2x is KSG. “Site” indicates the native, ungapped TIGR4 residue number, and “RefAA” denotes the corresponding TIGR4 reference amino acid. “Variation rate” was calculated as the number of nongap residues among the 204 local isolates that differed from TIGR4 divided by the effective number of nongap isolate residues at that site; TIGR4 itself was excluded from the denominator. “Minimum Cα–Cα distance (Å)” is the shortest distance from the candidate residue to any residue in the three motif sets.

### 2.7 Statistical analysis

Statistical analyses were performed in SPSS for Windows (version 22.0; Chicago, IL, USA) and with Python scripts. Categorical variables and proportions were compared using the chi-square test or Fisher’s exact test as appropriate. When an overall difference across multiple groups was observed, *post hoc* analyses were conducted using pairwise comparisons and one-vs.-rest 2 × 2 contingency testing where applicable, and *p* values were adjusted for multiple testing using Holm’s method. Effect sizes for contingency analyses were summarized using Cramér’s *V* when applicable. For nonparametric comparisons, the Mann–Whitney *U* test and the Kolmogorov–Smirnov test were applied. Continuous variables were analyzed using Student’s *t* test or one-way ANOVA, with correction for multiple comparisons where appropriate. All tests were two-sided, and *p* values <0.05 were considered to indicate statistical significance.

For the exploratory PBP–MIC analysis, PEN-MIC was treated as a seven-level ordered twofold-dilution outcome (0.12, 0.25, 0.5, 1, 2, 4, and ≥8 μg/mL), with ≥8 μg/mL treated as the highest censored category; a PEN MIC ≥4 μg/mL was used as a secondary descriptive high-MIC endpoint. Site-wise MIC distributions, exact PBP profiles, and non-TIGR4 residue burdens were evaluated using nonparametric tests as appropriate. Profiles represented by fewer than five isolates were pooled as rare profiles for omnibus testing. To assess confounding by clonal population structure, empirical *p* values were obtained from 20,000 GPSC-stratified permutations in which MIC values were shuffled only among isolates assigned to the same GPSC. Holm correction was applied within each analysis family.

## 3 Results

### 3.1 Demographic and clinical characteristics

**Figure 1:**
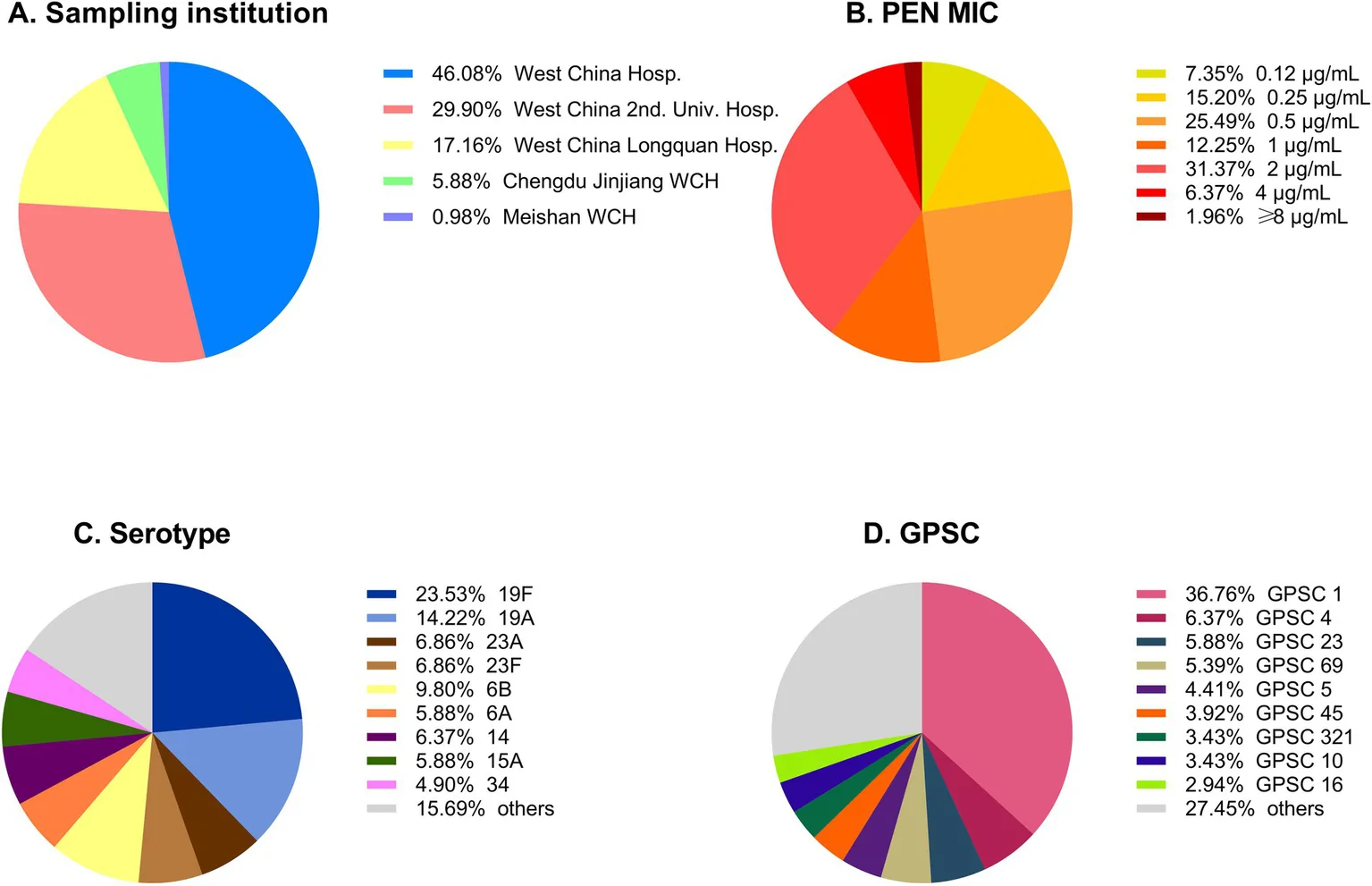
Overview of the 204 clinical isolates exhibiting reduced susceptibility to penicillin collected in Southwest China. Pie charts summarizing the distribution of **(A)** sampling institutions, **(B)** penicillin minimum inhibitory concentrations (PEN MIC, μg/mL), **(C)** molecular serotypes, and **(D)** global pneumococcal sequence clusters (GPSCs). The percentages indicate the percentages of isolates in each category; serotypes and GPSCs not listed individually are grouped as “others”.

A total of 204 clinical *S. pneumoniae* isolates collected from 5 institutions were included in this study (Figure 1A, Table 2). The isolates were obtained from 127 male (62.3%) and 77 female (37.7%) patients. Most isolates were recovered from patients aged ≤3 years (77, 37.7%) or 50–95 years (74, 36.3%), with a median (P25–P75) patient age of 5 (2–62) years. The majority of the isolates were recovered from Han (Chinese) individuals (194, 95.1%). Clinically, 51 isolates (25.0%) were associated with invasive pneumococcal disease, including meningitis (14, 6.9%) and sepsis (37, 18.1%), whereas 153 isolates (75.0%) were associated with noninvasive pneumococcal disease, predominantly pneumonia (148, 72.5%). With respect to specimen sources, the isolates were recovered mainly from sputum (122, 59.8%), followed by blood (37, 18.1%) and cerebrospinal fluid (14, 6.9%), with smaller proportions recovered from chest drainage, alveolar lavage fluid, tracheal secretions, and other specimen types.

**Table 2:** The demographic and clinical characteristics of the patients from whom the 204 clinical isolates exhibiting reduced susceptibility to penicillin were collected.

Characteristic	No. of isolates	(%)
Sex		
Male	127	62.3
Female	77	37.7
Age (years)		
≤3	77	37.7
4–17	39	19.1
18–49	14	6.9
50–95	74	36.3
*Median age (P25-P75)*	*5 (2–62)*	
Ethnicity		
Han (Chinese)	194	95.1
Tibetan (Chinese)	9	4.4
Han (Singaporean)	1	0.5
Diagnosis		
Invasive pneumococcal disease (IPD)	51	25.0
Meningitis	14	6.9
Sepsis	37	18.1
Noninvasive pneumococcal disease (NIPD)	153	75.0
Pneumonia	148	72.5
Otitis media	2	1.0
Urinary tract infection	1	0.5
Other localized infections	2	1.0
Sample type		
Cerebrospinal fluid	14	6.9
Blood	37	18.1
Chest drainage	7	3.4
Alveolar lavage fluid	11	5.4
Tracheal secretions	8	3.9
Sputum	122	59.8
Ear canal secretions	2	1.0
Midstream urine	1	0.5
Pus	2	1.0
*Total isolates*	*204*	*100.0*

### 3.2 Antibiotic susceptibility

All 204 clinical *S. pneumoniae* isolates included in this study were selected on the basis of reduced susceptibility to penicillin, defined as a penicillin MIC ≥0.12 μg/mL, with the CLSI meningitis breakpoint used as a screening threshold. Accordingly, when the meningitis breakpoint was used for interpretation, all the isolates were categorized as penicillin resistant. The penicillin MIC₅₀ and MIC₉₀ were 1 and 2 μg/mL, respectively. The penicillin MIC distribution was dominated by MICs of 0.5–2 μg/mL, with 2 μg/mL as the most frequently measured concentration (64/204, 31.37%); only a small proportion of the isolates had a high MIC (≥8 μg/mL: 4/204, 1.96%) (Figure 1B).

**Figure 2:**
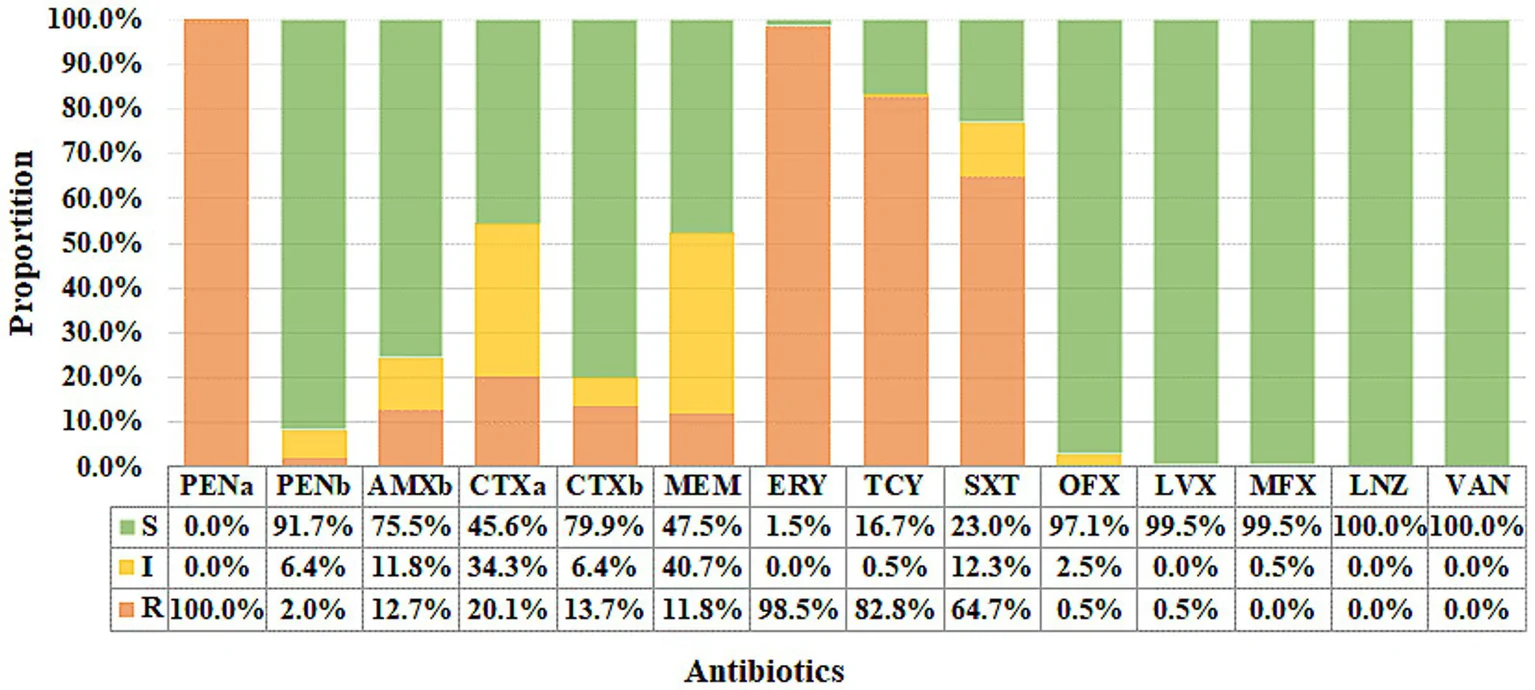
Antimicrobial susceptibility profiles of the 204 clinical isolates exhibiting reduced susceptibility to penicillin collected in Southwest China. The stacked bars show the proportions of susceptible (S), intermediate (I), and resistant (R) isolates for each antimicrobial agent, interpreted according to CLSI criteria. Penicillin and selected β-lactams are presented using two breakpoint frameworks: a, meningitis breakpoints; b, nonmeningitis breakpoints. As one of the inclusion criteria in this study was a PEN MIC ≥ 0.12 μg/mL, all the isolates were categorized as resistant when interpreted with respect to the meningitis penicillin breakpoint (PENa). PEN, penicillin; AMX, amoxicillin; CTX, cefotaxime; MEM, meropenem; ERY, erythromycin; TCY, tetracycline; SXT, trimethoprim–sulfamethoxazole; OFX, ofloxacin; LVX, levofloxacin; MXF, moxifloxacin; LNZ, linezolid; VAN, vancomycin.

For nonpenicillin agents, the most prominent finding was the high prevalence of resistance to erythromycin (98.5%) and tetracycline (82.8%), along with the substantial prevalence of resistance to trimethoprim–sulfamethoxazole (64.7%). In contrast, susceptibility to fluoroquinolones remained largely preserved (≥97% susceptibility rate), and no resistance to vancomycin or linezolid was detected (100% susceptibility rate for both). Overall, these results indicate that among the isolates with elevated penicillin MICs, macrolide and tetracycline resistance was highly prevalent, whereas glycopeptide/oxazolidinone activity and the activity of most fluoroquinolones were maintained (Figure 2).

### 3.3 Molecular serotype distribution

Among the 204 clinical isolates we collected in Southwest China, 27 distinct serotypes were identified, including one unresolved 24B/24C/24F assignment (Figure 1C). The serotypes most frequently detected were 19F (48/204, 23.5%), 19A (29/204, 14.2%), 6B (20, 9.8%), 23A (14/204, 6.9%), 23F (14/204, 6.9%), 14 (13, 6.4%), 6A (12/204, 5.9%) and 15A (12/204, 5.9%). On the basis of the serotype composition, the estimated PCV13 coverage was 69.6% (142/204), and the estimated PPV23 coverage was 65.2% (133/204) (PPV23 does not include serotype 6A, which is included in PCV13).

As of July 31, 2025, the PubMLST Pneumococcal Genome Library contained 34,108 records of *S. pneumoniae* isolates, of which 10,729 included reported penicillin minimum inhibitory concentration (MIC) data. Applying the predefined criterion for isolates exhibiting reduced susceptibility to penicillin (penicillin MIC ≥0.12 μg/mL), 3,945 records were eligible for inclusion. Genome assemblies (FASTA files) were retrieved for all eligible records; after reprocessing through our quality control pipeline and reannotation with Prokka, 3,611 assemblies passed QC and were retained for downstream analyses. Notably, these 3,611 PubMLST isolates were contributed predominantly by the United States and Thailand (3,064/3611, 84.85%) and included 1748 isolates from the United States (48.41%) and 1,316 isolates from Thailand (36.44%).

**Figure 3:**
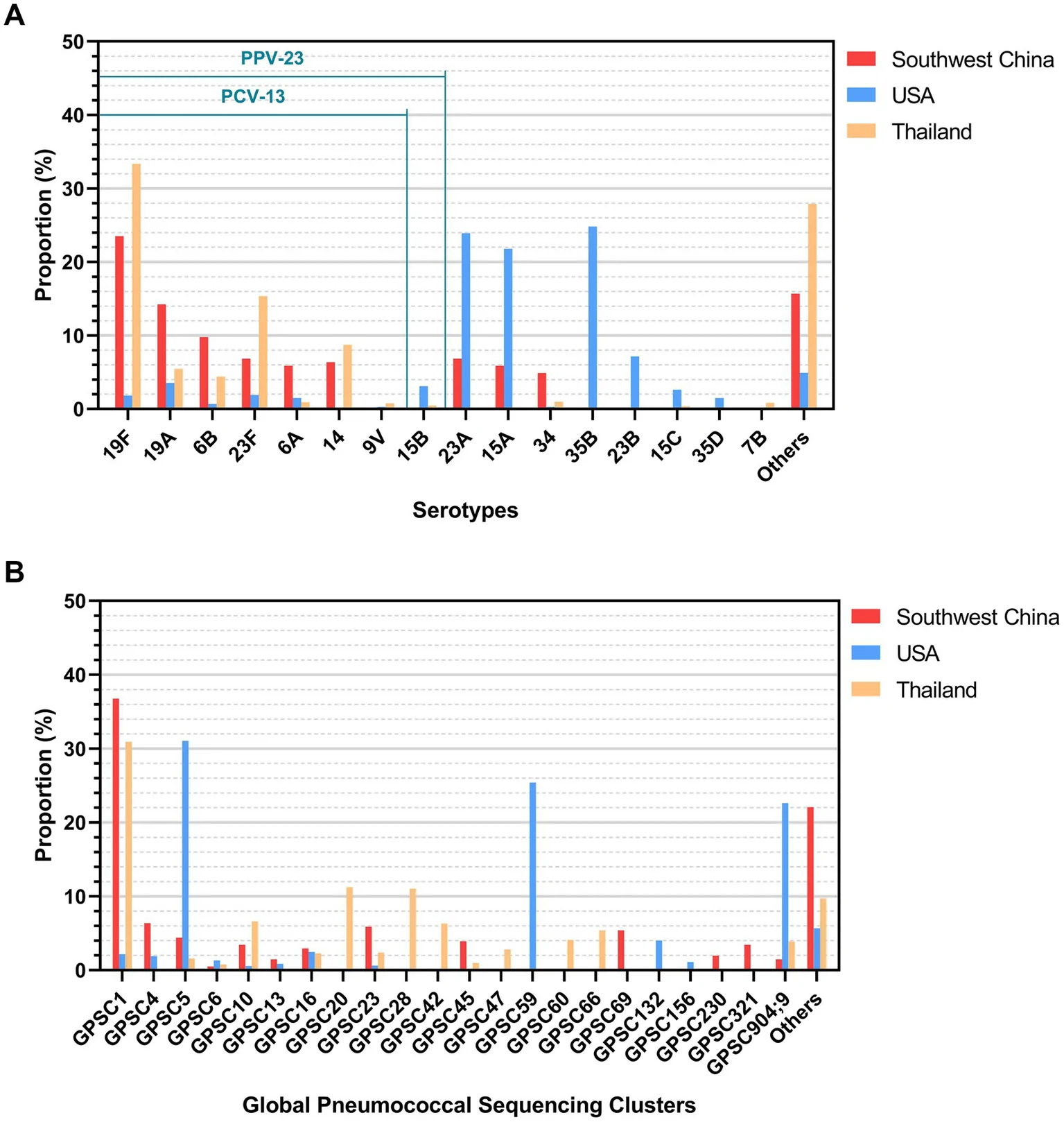
Comparison of serotype and GPSC compositions among *Streptococcus pneumoniae* isolates exhibiting reduced susceptibility to penicillin originating from Southwest China, the United States, and Thailand. The **(A)** serotype composition and **(B)** Global Pneumococcal Sequence Cluster (GPSC) composition are shown as grouped bar charts. The isolates from Southwest China were collected by our study team (*n* = 204), whereas the isolates from the United States (*n* = 1,748) and Thailand (*n* = 1,316) were retrieved from PubMLST. The most frequently detected serotypes differed across the countries: Southwest China—19F, 19A, and 6B; the United States—35B, 23A, and 15A; and Thailand—19F, 23F, and 19A. The most common GPSC also varied by country, with GPSC1 as the dominant lineage in both Southwest China and Thailand, whereas GPSC5 predominated in the United States. The horizontal brackets in panel **(A)** are schematic visual guides to vaccine-associated serotypes and should not be interpreted as inclusive intervals across the *x*-axis; specifically, serotype 6A contributes to PCV13 but not PPV23 coverage.

The serotype composition differed substantially by country (Figure 3A). Among the isolates from the United States, the most frequently detected serotypes were 35B (434/1748, 24.8%), 23A (418/1748, 23.9%), 15A (381/1748, 21.8%) and 23B (125/1748, 7.2%). In contrast, among the isolates from Thailand, the most frequently detected types were 19F (439/1316, 33.4%), 23F (202/1316, 15.3%), 14 (115/1316, 8.7%) and 19A (72/1316, 5.5%).

The overall serotype composition differed significantly among Southwest China, the United States and Thailand (*χ*^2^ = 2452.85, *p* < 0.001; Cramér’s *V* = 0.613). Pairwise comparisons further confirmed the marked between-country heterogeneity (Holm-adjusted *p* < 0.001 for each pair). Compared with the United States, Southwest China had higher percentages of isolates belonging to serotypes 19F (23.5% vs. 1.8%, *p*_adj < 0.001), 19A (14.2% vs. 3.5%, *p*_adj < 0.001) and 6B (9.8% vs. 0.7%, *p*_adj < 0.001), whereas isolates from the United States were enriched for 35B (24.8% vs. 2.5%, *p*_adj < 0.001), 23A (23.9% vs. 6.9%, *p*_adj < 0.001) and 15A (21.8% vs. 5.9%, *p*_adj < 0.001). Compared with Thailand, notable excesses in Southwest China were observed for serotypes 19A (14.2% vs. 5.5%, *p*_adj < 0.001), 6B (9.8% vs. 4.4%, *p*_adj = 0.028), 23A (6.9% vs. 0.1%, *p*_adj < 0.001), 15A (5.9% vs. 0.1%, *p*_adj < 0.001) and 6A (5.9% vs. 0.9%, *p*_adj < 0.001), whereas Thailand had higher percentages of serotypes 23F (15.3% vs. 6.9%, *p*_adj = 0.008) and 19F (33.4% vs. 23.5%, *p*_adj = 0.038). The direct comparison between the United States and Thailand similarly showed strong divergence, including enrichment of serotypes 35B, 23A and 15A in the United States and serotypes 19F, 23F and 14 in Thailand (all Holm-adjusted *p* < 0.001) (Supplementary Table 1).

In terms of theoretical vaccine serotype coverage, Southwest China differed markedly from the United States. The percentage of isolates assigned to PCV13 serotypes was higher in Southwest China than in the United States (69.6% vs. 10.0%, *p*_adj < 0.001); similarly, the percentage assigned to PPV23 serotypes was higher in Southwest China (65.2% vs. 13.7%, *p*_adj < 0.001). In contrast, Southwest China and Thailand had comparable estimated coverage for both PCV13 (69.6% vs. 69.0%, *p*_adj = 0.935) and PPV23 (65.2% vs. 68.9%, *p*_adj = 0.293) (Supplementary Table 1).

Because the PubMLST genomes included here were selected under the same predefined threshold for reduced penicillin susceptibility, the above comparisons reflect differences in the serotype composition among the subsets enriched with isolates exhibiting reduced penicillin susceptibility across countries rather than estimates of the background serotype prevalence.

### 3.4 GPSC distribution

Among the 204 clinical *S. pneumoniae* isolates that exhibited reduced susceptibility to penicillin, 74 distinct sequence types were identified by multilocus sequence typing. The most frequently detected sequence types were ST271 (33/204, 16.2%) and ST320 (31/204, 15.2%), followed by ST876 (10/204, 4.9%), ST90 (10/204, 4.9%) and ST11972 (9/204, 4.4%).

Using the Global Pneumococcal Sequence Cluster (GPSC; https://www.pneumogen.net/gps/) scheme, which defines internationally comparable pneumococcal lineages on the basis of whole-genome relatedness, 190 isolates were assigned to 29 GPSCs, whereas 14 isolates could not be confidently assigned. The collection was dominated by GPSC1 (75/204, 36.8%), with additional contributions from GPSC4 (13/204, 6.4%), GPSC23 (12/204, 5.9%) and GPSC69 (11/204, 5.4%) (Figure 1D). Overall, the MLST sequence types showed a stable nested relationship within the GPSCs in this dataset, with each major ST mapping predominantly to a single GPSC and each GPSC comprising a limited set of related STs (Supplementary Table 2).

Within the PubMLST dataset passing all steps in our filtering and quality control pipeline, isolates from the United States (*n* = 1748) and Thailand (*n* = 1,316) collectively accounted for 3064/3611 (84.8%) genomes (Figure 3B). The isolates from the United States were dominated by GPSC5 (543/1748, 31.1%), GPSC59 (444/1748, 25.4%), and GPSC904;9 (395/1748, 22.6%), whereas GPSC1 contributed only 38/1748 (2.2%) isolates. In contrast, the isolates from Thailand were assigned primarily to GPSC1 (407/1316, 30.9%), followed by GPSC20 (148/1316, 11.2%), GPSC28 (145/1316, 11.0%), GPSC10 (87/1316, 6.6%) and GPSC42 (83/1316, 6.3%).

The overall GPSC composition differed significantly among Southwest China, the United States, and Thailand (*χ*^2^ = 3595.72, *p* < 0.001; Cramér’s *V* = 0.742). Pairwise comparisons further confirmed the marked between-country heterogeneity (Holm-adjusted *p* < 0.001 for each pair). Compared with the United States, Southwest China had higher percentages of isolates belonging to GPSC1 (36.8% vs. 2.2%, *p*_adj < 0.001), GPSC4 (6.4% vs. 1.9%, *p*_adj = 0.007) and GPSC23 (5.9% vs. 0.6%, *p*_adj < 0.001), whereas the isolates from the United States were enriched for GPSC5 (31.1% vs. 4.4%, *p*_adj < 0.001), GPSC59 (25.4% vs. 0.0%, *p*_adj < 0.001) and GPSC904;9 (22.6% vs. 1.5%, *p*_adj < 0.001). In comparison with Thailand, Southwest China had higher percentages of isolates belonging to GPSC4 (6.4% vs. 0.0%, *p*_adj < 0.001), GPSC69 (5.4% vs. 0.0%, *p*_adj < 0.001) and GPSC321 (3.4% vs. 0.0%, *p*_adj < 0.001), whereas Thailand had higher percentages of isolates belonging to GPSC20 (11.2% vs. 0.0%, *p*_adj < 0.001), GPSC28 (11.0% vs. 0.0%, *p*_adj < 0.001) and GPSC42 (6.3% vs. 0.0%, *p*_adj < 0.001). Notably, despite these differences, Southwest China and Thailand shared a broadly similar lineage structure in that GPSC1 was the most common lineage in both regions (36.8 and 30.9%, respectively) (Supplementary Table 1).

### 3.5 Phylogenetic analysis

**Figure 4:**
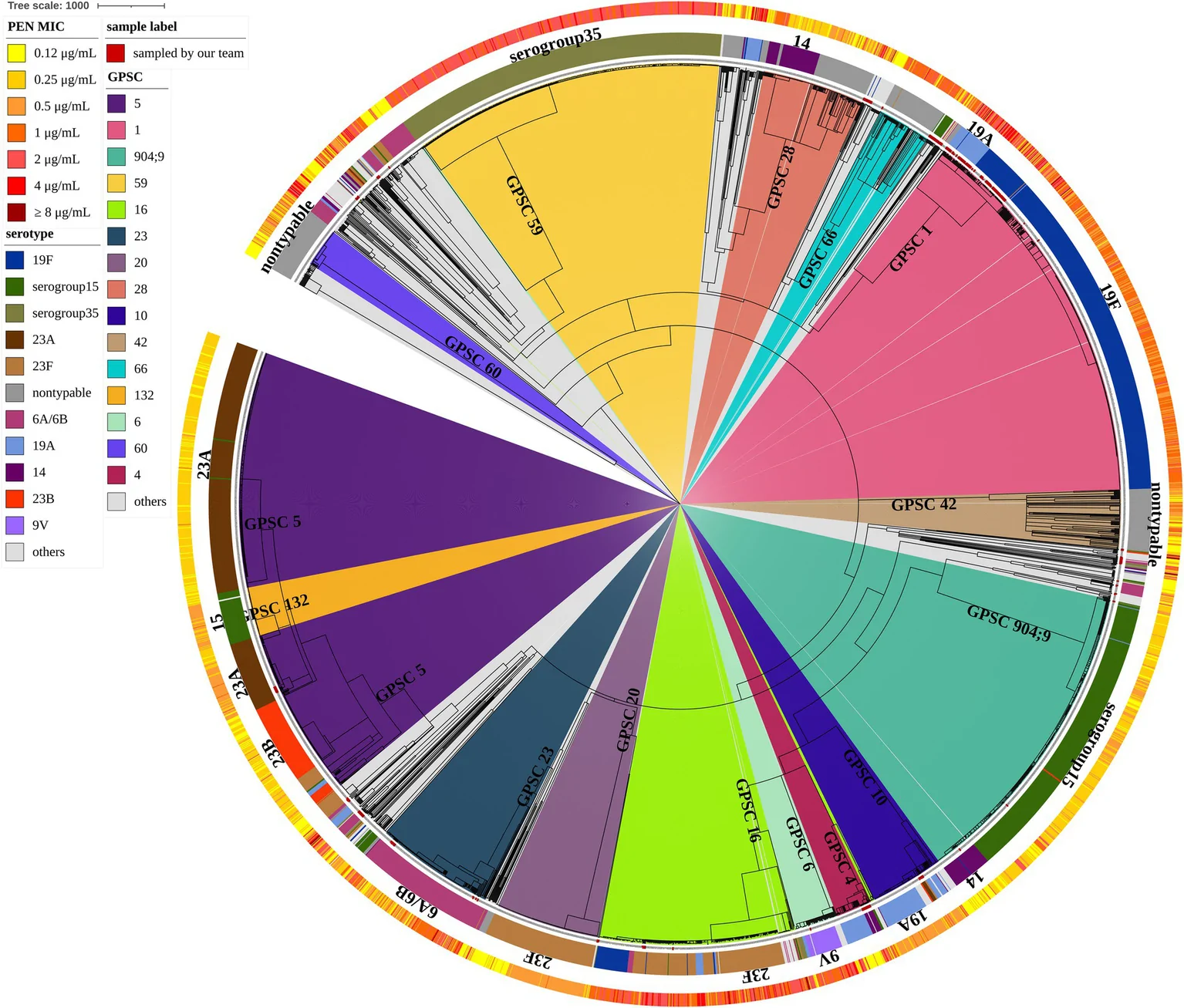
Core genome phylogeny of *Streptococcus pneumoniae* isolates exhibiting reduced susceptibility to penicillin. A maximum likelihood tree was constructed using the 204 clinical isolates collected in Southwest China, 3,611 PubMLST genomes passing the filtering/QC pipeline used in the study, and the TIGR4 reference genome. From the inner ring to the outer ring, the isolates are annotated by their GPSC assignment, sample label (study-collected vs. PubMLST-derived), molecular serotype, and penicillin MIC category. The phylogeny shows pronounced clustering by GPSC and serotype, indicating strong lineage structuring across the combined dataset.

The core genome phylogenetic tree constructed from the PubMLST-derived genomes (*n* = 3,611) together with our collected isolates (*n* = 204) exhibited a pronounced lineage structure, with major branches forming well-demarcated clusters corresponding to Global Pneumococcal Sequence Clusters (GPSCs) (Figure 4). Across the phylogeny, the GPSC assignments were largely concordant with the clade-level clustering. The serotype annotation showed clear, nonrandom aggregation along these major lineages, with several dominant serotype–GPSC associations observed at the clade level. Specifically, GPSC1 was associated mainly with serotypes 19F and 19A, whereas GPSC5 clustered largely with serotypes 23A and 23B. GPSC132 was linked predominantly to serogroup 15, GPSC23 was associated mainly with serotypes 6A/6B, and GPSC59 aligned closely with serogroup 35. In addition, serotype 23F was clustered primarily within GPSC20 and GPSC16, whereas GPSC904;9 consisted primarily of serogroup 15 with a minor segment of serotype 14. Notably, the lineages assigned to GPSC60 and GPSC42 were almost exclusively capsular nontypable strains in this dataset.

Although the penicillin MIC categories were intermingled across the phylogeny and were not confined to a single lineage or serotype, several dominant trends were observed. At the GPSC level, some lineages exhibited a relatively restricted MIC range—for example, GPSC5 and GPSC904;9, in which high MIC values (defined here as ≥4 μg/mL) were rare or absent (0/581 and 0/452, respectively). In contrast, GPSC16 had a broader MIC spectrum and a greater proportion of isolates with a MIC ≥4 μg/mL (49/284, 17.3%; Holm-adjusted *p* < 0.001 vs. the remainder of the isolates), whereas GPSC1 displayed an intermediate pattern (41/557, 7.4%; *p*_adj = 0.033). GPSC59, largely corresponding to serogroup 35, was dominated by a MIC of approximately 2 μg/mL, with a smaller proportion of MICs of 4 μg/mL (26/444, 5.9%), while GPSC23 (comprising mainly serotypes 6A/6B) was dominated by isolates with MICs in the 0.5–1 μg/mL range but contained some isolates with higher MICs, including a small number of isolates with a MIC ≥8 μg/mL. Consistent with the serotype track, lineages dominated by capsular nontypable strains (e.g., GPSC60 and GPSC42) generally presented lower MICs (almost exclusively ≤2 μg/mL).

At the serotype level, the association with penicillin MIC appeared weaker and overlapped substantially across groups. Nevertheless, serotype 19A contained a significantly greater proportion of isolates with an MIC ≥4 μg/mL (41/216, 19.0%; *p*_adj < 0.001). By contrast, none of the 433 serotype 23A isolates had an MIC ≥4 μg/mL (0/433, 0.0%), a proportion significantly lower than that among all other serotypes combined (*p*_adj < 0.001). Serotype 23F showed a heterogeneous MIC distribution (MIC ≥4 μg/mL: 36/411, 8.8%; *p*_adj = 0.002), consistent with its distribution across multiple GPSCs with different MIC profiles.

### 3.6 Virulence genes and antibiotic resistance-related genes

Virulence and resistance determinants were assessed in the 204 clinical isolates collected in Southwest China with reduced susceptibility to penicillin. The VFDB screen revealed several core virulence loci, including *nanA, psaA, pavA, pce/cbpE, ply* and *lytA* (each ≥98%), in nearly all the isolates. In contrast, multiple colonization-associated loci, including the pilus-1 operon (33.3% overall), pilus-2 operon (36.8%), *pfbA* (61.8%), *cbpG* (38.2%), *iga* (42.6%) and *nanB* (90.7%), exhibited marked heterogeneity across the collection. Importantly, the detection profiles of these variable loci were strongly structured by the GPSC background (all Holm-adjusted *p* ≤ 0.002; Table 3). For example, the pilus-1 operon was markedly more common in the GPSC1 isolates than in the non-GPSC1 isolates (66/75, 88.0% vs. 2/129, 1.6%), and the pilus-2 operon was detected in all the GPSC1 isolates (75/75, 100.0%) but in none of the non-GPSC1 isolates (0/129, 0.0%). Conversely, *pfbA* showed the opposite pattern (0/75, 0.0% in GPSC1 isolates vs. 126/129, 97.7% in non-GPSC1 isolates). Similarly, *iga* was strongly enriched in GPSC1 isolates (75/75, 100.0% vs. 12/129, 9.3%), and *cbpG* was largely undetected in GPSC1 isolates but frequently detected in non-GPSC1 isolates (0/75, 0.0% vs. 78/129, 60.5%). In addition, *nanB* was detected in all the GPSC1 isolates (75/75, 100.0%) but was detected less consistently in the non-GPSC1 isolates (110/129, 85.3%).

**Table 3:** Distribution of virulence and antimicrobial resistance-related genes in the 204 clinical isolates exhibiting reduced susceptibility to penicillin collected in Southwest China.

Gene	Overall	GPSC1	Non-GPSC1	*p*
Virulence-related				
*ply*	204 (100.0%)	75 (100.0%)	129 (100.0%)	1.000
*pavA*	204 (100.0%)	75 (100.0%)	129 (100.0%)	1.000
*pfbA*	126 (61.8%)	0 (0.0%)	126 (97.7%)	**<0.001**
*cbpD*	190 (93.1%)	73 (97.3%)	117 (90.7%)	0.702
*cbpG*	78 (38.2%)	0 (0.0%)	78 (60.5%)	**<0.001**
*pce/cbpE*	204 (100.0%)	75 (100.0%)	129 (100.0%)	1.000
*nanA*	204 (100.0%)	75 (100.0%)	129 (100.0%)	1.000
*nanB*	185 (90.7%)	75 (100.0%)	110 (85.3%)	**0.002**
*lytA*	202 (99.0%)	74 (98.7%)	128 (99.2%)	1.000
*hysA*	201 (98.5%)	74 (98.7%)	127 (98.4%)	1.000
*iga*	87 (42.6%)	75 (100.0%)	12 (9.3%)	**<0.001**
*psaA*	204 (100.0%)	75 (100.0%)	129 (100.0%)	1.000
Pilus-1 operon	68 (33.3%)	66 (88.0%)	2 (1.6%)	**<0.001**
Pilus-2 operon	75 (36.8%)	75 (100.0%)	0 (0.0%)	**<0.001**
Antibiotic resistance-related				
Macrolide (*ermB*)	200 (98.0%)	74 (98.7%)	126 (97.7%)	1.000
Macrolide (*mefA*)	82 (40.2%)	75 (100.0%)	7 (5.4%)	**<0.001**
Tetracycline (*tet*)	201 (98.5%)	75 (100.0%)	126 (97.7%)	**0.598**
Co-trimoxazole (*folA*)	143 (70.1%)	74 (98.7%)	69 (53.5%)	**<0.001**
Co-trimoxazole (*folP*)	162 (79.4%)	75 (100.0%)	87 (67.4%)	**<0.001**
Chloramphenicol (*cat*)	16 (7.8%)	0 (0.0%)	16 (12.4%)	**0.003**
Fluoroquinolone QRDR mutations (*gyr/par*)	7 (3.4%)	0 (0.0%)	7 (5.4%)	0.146

Values are presented as *n* (%); the denominators were 204 isolates overall, 75 GPSC1 isolates, and 129 non-GPSC1 isolates. For each determinant, a two-sided Fisher’s exact test was applied to the 2 × 2 table defined by GPSC1 status and determinant detection. Holm correction was applied separately within the virulence-related and antimicrobial resistance-related families, and the P column reports the adjusted values. Pilus-1 operon positivity required detection of all six listed components, whereas Pilus-2 operon positivity required detection of all five listed components at the predefined ≥80% identity threshold. Mef(A) and msr(D) were co-detected in the same 82 isolates. Bold values indicate statistically significant Holm-adjusted *p* values (*p* < 0.05).

Antimicrobial resistance determinants were common in this cohort with reduced penicillin susceptibility. Macrolide resistance determinants were nearly universally present (*ermB*, 200/204; 98.0%), whereas *mefA* was detected in 82/204 (40.2%) isolates. Tetracycline determinants (*tet*) were detected in 201/204 (98.5%) isolates. Co-trimoxazole determinants were frequently detected (*folA*, 143/204, 70.1%; *folP*, 162/204, 79.4%). In contrast, chloramphenicol (*cat*, 16/204, 7.8%) and fluoroquinolone QRDR mutations (*gyr/par*, 7/204, 3.4%) were uncommon. The frequencies of several resistance determinants differed significantly by GPSC background (Table 3): compared with the non-GPSC1 isolates, the GPSC1 isolates had markedly higher detection rates of *mefA* (75/75, 100.0% vs. 7/129, 5.4%; Holm-adjusted *p < 0.001*) and co-trimoxazole determinants (*folA*: 74/75, 98.7% vs. 69/129, 53.5%, Holm-adjusted *p* < 0.001; *folP*: 75/75, 100.0% vs. 87/129, 67.4%, Holm-adjusted *p* < 0.001), whereas *cat* was not detected in any GPSC1 isolate but was detected in 16/129 (12.4%) non-GPSC1 isolates (Holm-adjusted *p* = 0.003). In contrast, *ermB* and *tet* exhibited comparable detection rates in the GPSC1 and non-GPSC1 isolates (both Holm-adjusted *p* > 0.05).

**Figure 5:**
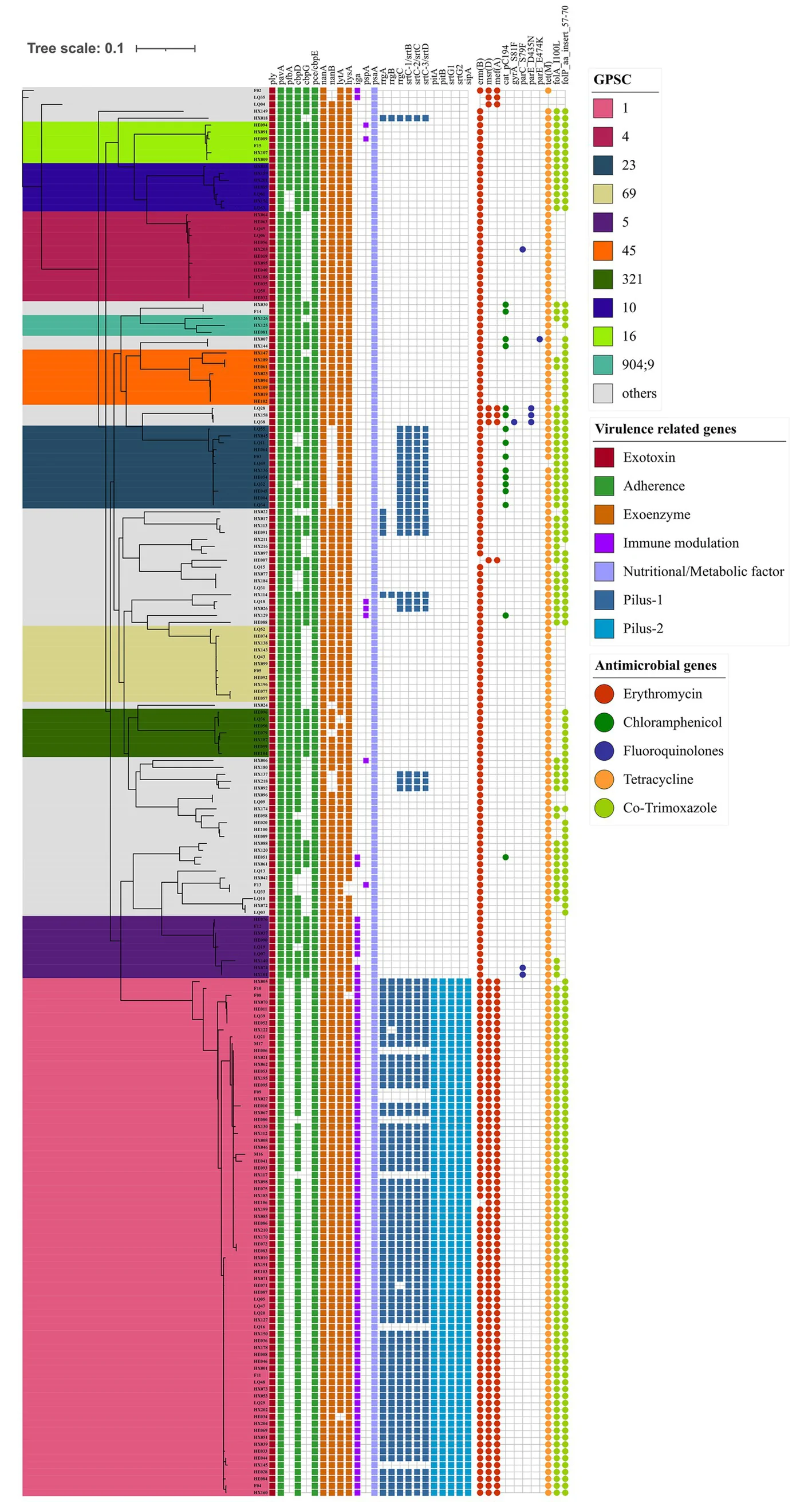
Core genome phylogeny of the 204 clinical *Streptococcus pneumoniae* isolates exhibiting reduced susceptibility to penicillin collected in Southwest China, shown in a rectangular layout and annotated with virulence- and antimicrobial resistance-related genes. The tree tips are aligned with the annotation rows, and the colored clade backgrounds indicate GPSC assignments. The rectangular columns to the right of the tree summarize the presence or absence of individual virulence-related genes and antimicrobial resistance determinants, with colors corresponding to the functional categories shown in the legend. Gene presence was defined as meeting an identity cutoff of ≥80%. Overall, the annotation matrix shows lineage-structured, GPSC-aligned patterns rather than a random distribution across the phylogeny.

As visualized in the phylogeny (Figure 5), both virulence and resistance-related genes displayed clear lineage-structured patterns rather than a random distribution across the tree. Variable colonization-associated loci formed discrete, GPSC-aligned blocks: the pilus-1 and pilus-2 signals were clustered almost exclusively within the GPSC1 clade, whereas *pfbA* and *cbpG* were largely confined to non-GPSC1 backgrounds. Similarly, antimicrobial determinants annotated in the corresponding rectangular presence/absence tracks showed GPSC-dependent aggregation, with the *mefA* and f*olA/folP* signals concentrated within GPSC1, whereas *cat* and the rare QRDR mutations appeared only sporadically and were restricted to non-GPSC1 branches. Collectively, the annotation tracks emphasize that the observed heterogeneity is explained primarily by the phylogenetic background, a finding consistent with the GPSC-stratified comparisons summarized in Table 3.

### 3.7 Penicillin-binding protein analysis

**Figure 6:**
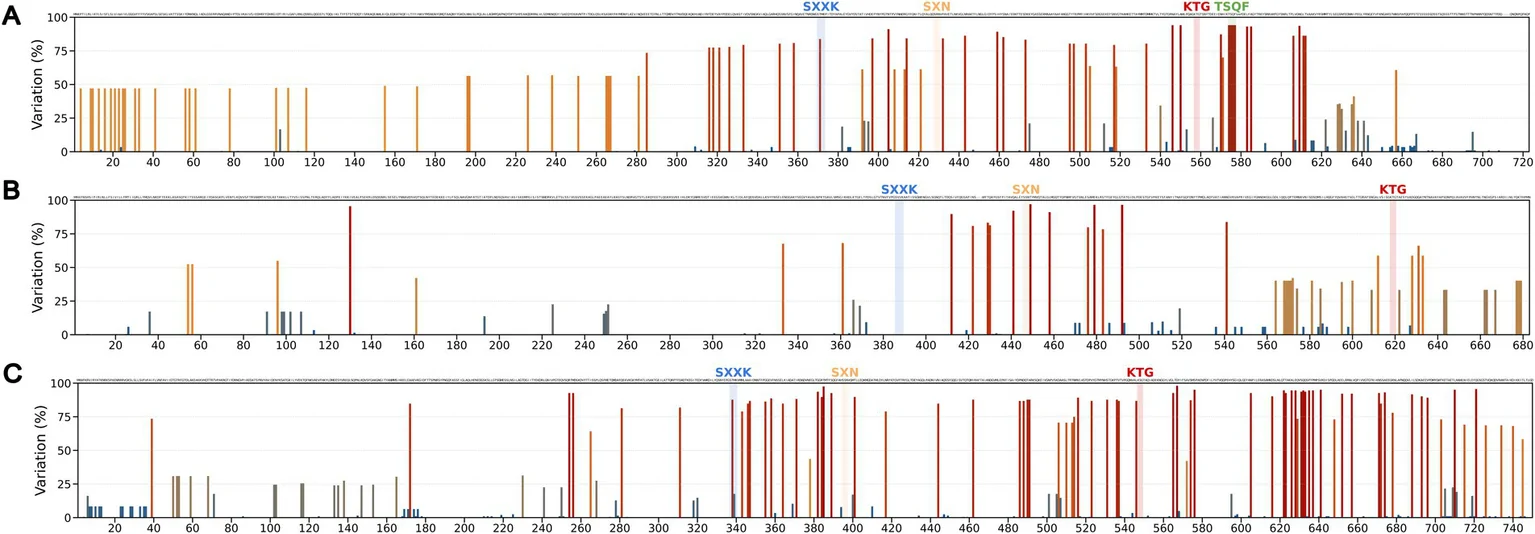
Site-specific amino acid variation profiles of penicillin-binding proteins in clinical *Streptococcus pneumoniae* isolates. Linear bar plots showing the per-site amino acid variation rates for **(A)** PBP1a, **(B)** PBP2b, and **(C)** PBP2x in the 204 clinical *Streptococcus pneumoniae* isolates exhibiting reduced susceptibility to penicillin collected in Southwest China. The height of each bar represents the proportion of the remaining isolate residues that differed from the corresponding TIGR4 residue. Bars are colored according to variation frequency along a gradient from low (blue) through intermediate (orange) to high (red) variability. The bars and numeric x-axes use one-based MAFFT alignment-column coordinates, thereby retaining columns introduced by insertions and deletions, whereas the motif labels and shaded guides are referenced to the native, ungapped TIGR4 residue-numbering scheme and are therefore schematic where indel columns occur. In panel B, MAFFT alignment columns 424–426 are gaps in TIGR4 and accommodate a three-residue insertion in a subset of isolates. For consistency across panels, “KTG” is used in panel C as the conventional generic label for the third conserved PBP catalytic motif; the corresponding native motif in TIGR4 PBP2x is KSG, represented collectively in the text as K(T/S)G.

To characterize PBP sequence variation among clinical *S. pneumoniae* isolates with reduced susceptibility to penicillin in Southwest China, we extracted the *pbp1a, pbp2b*, and *pbp2x* sequences from the 204 isolates and quantified site-specific amino acid variability. Throughout the text, all residue positions are reported using the native TIGR4 numbering system. The resulting linear variation profiles (Figure 6) revealed that substitutions were unevenly distributed and clustered predominantly within the C-terminal transpeptidase region and the sequences surrounding the conserved catalytic motifs [SXXK, SXN, and K(T/S)G (KTG)], a pattern consistent with focal remodeling of the β-lactam binding environment.

In PBP1a (Figure 6A), 20.0% (144/719) of highly supported positions exhibited variability, with several nearly fixed hotspots. The greatest changes in frequency were detected for N546G and A550P (both 94.6%), as well as for the canonical resistance-associated TSQF (574–577) block substitution (T574N, S575T, Q576G, and F577Y, each 94.6%), indicating that the TSQF region was widely altered in this collection. Within the SXXK (STMK) motif, the nucleophilic residues S370 and K373 were fully conserved, whereas the second residue showed marked divergence (T371, 84.3%; predominantly T → S/A), highlighting the strong selection at the motif-adjacent position. Additional highly variable sites in the top ~3% included N609D (94.1%), L583M (93.6%), and A585V (93.6%) (Supplementary Table 3).

In PBP2b (Figure 6B), the overall variability was lower (14.1%, 96/680 variable positions), and the catalytic motifs were strongly conserved (SXXK: 386–389, SXN: 443–445, and KTG: 615–617 showed no substitutions at the conserved residues). The high-frequency substitutions were instead concentrated in nearby nonmotif positions; the T446A/S (97.5%), E476G (97.1%), and T489A/S/V (97.1%) substitutions were dominant, with additional recurrent substitutions such as I130T (96.1%), Q438E (92.6%), L455I (91.7%), and S412P (90.2%), suggesting extensive remodeling surrounding—but not within—the catalytic core (Supplementary Table 3).

In PBP2x (Figure 6C), the variability was the greatest (25.1%, 188/750 variable positions), with dense clustering across the transpeptidase region and the C-terminal segment. The most frequent substitutions included D567N (98.5%) and T385M (98.0%), and multiple nearly fixed changes in the distal C-terminus (e.g., Q721E 96.1%, L710F 95.6%, and V641I 95.6%) were detected. Notably, PBP2x also showed pronounced divergence within the SXXK region: S337 and K340 remained conserved but the second residue, T338, displayed a high substitution rate (88.2%, mainly T → A), and adjacent positions exhibited additional variability, paralleling the pattern observed for PBP1a. In summary, PBP1a and PBP2x shared prominent SXXK-adjacent divergence, whereas PBP2b exhibited comparatively conserved catalytic motifs with hotspots concentrated in surrounding regions. This distribution was consistent with coordinated, motif-proximal remodeling across multiple PBPs without disruption of the essential catalytic residues, but it did not by itself demonstrate that these substitutions independently determined the penicillin MIC (Supplementary Table 3).

**Figure 7:**
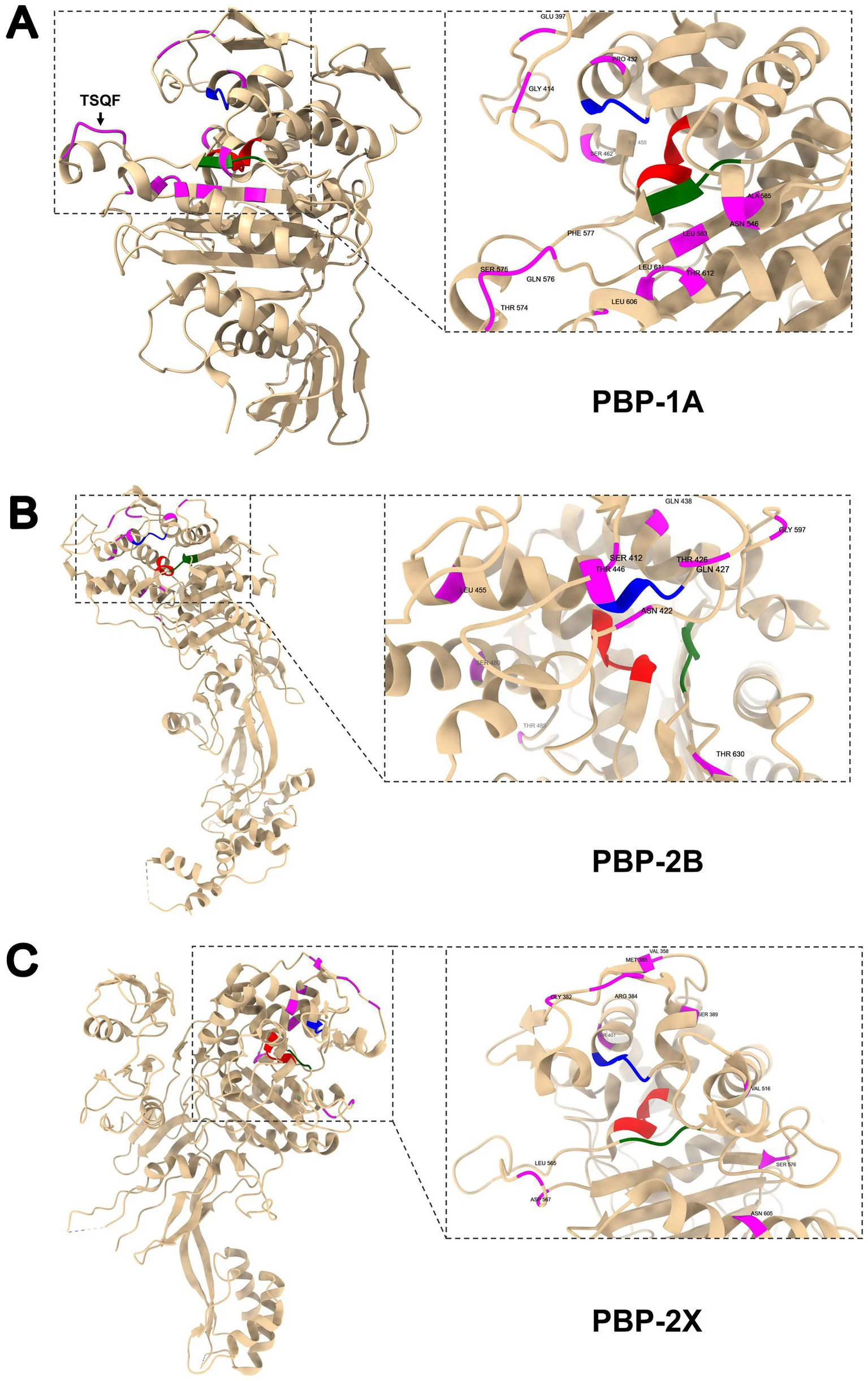
Structural mapping of candidate PBP substitutions in clinical *Streptococcus pneumoniae* isolates exhibiting reduced susceptibility to penicillin collected in Southwest China **(A)** PBP1a structure (PDB: 2C6W) **(B)** PBP2b structure (PDB: 2WAF) **(C)** PBP2x structure (PDB: 5OIZ). For each protein, the left panel shows the overall folded structure, and the dashed box indicates the catalytic/transpeptidase region shown in the enlarged view on the right. The candidate sites prioritized from the sequence variability analysis are highlighted in magenta. The three conserved active site motifs are colored as follows: SXXK (blue), SXN (green), and K(T/S)G (red). In PBP1a, the TSQF region is additionally indicated. Overall, the candidate sites in PBP1a, PBP2b, and PBP2x are predominantly concentrated within (or immediately adjacent to) the transpeptidase domain and are frequently located in spatial proximity to the conserved catalytic motifs, a pattern consistent with a potential contribution to reduced β-lactam susceptibility.

Building on the per-site variability profiles, we next prioritized mutations with a high likelihood of perturbing β-lactam binding by integrating the sequence variability with the three-dimensional spatial proximity to the catalytic center. We applied a structure-guided filter that retained only noncatalytic residues fulfilling two criteria: a variation rate at or above the protein-specific 95th percentile among high-support, structurally mappable noncatalytic positions and a minimum Cα–Cα distance of ≤15 Å from a catalytic-motif residue within the SXXK, SXN, or K(T/S)G motif. The conserved catalytic motifs surrounding the active site cleft were highlighted, and the final noncatalytic candidate residues were overlaid (Figure 7). This approach resulted in the identification of 12 candidate sites for PBP1a, 11 for PBP2b, and 11 for PBP2x (Table 1), all of which were located on surface-exposed loops or helices surrounding the catalytic pocket rather than at the essential catalytic residues themselves.

In PBP1a (Figure 7A), the candidate set formed a dense spatial cluster adjacent to the active site cleft and notably included Phe577, located within the classical resistance-associated TSQF region. This region, which showed nearly fixed substitutions in the linear variability analysis, thus also met the structural proximity criterion. Additional candidates in PBP1a were distributed in the region surrounding the catalytic pocket and included residues near positions 397/414/432/459/462 and a contiguous segment spanning the sequence approximately comprising positions 546–612, forming a coherent shell surrounding the SXXK, SXN, and KTG motifs.

In PBP2b (Figure 7B), the candidate residues were similarly concentrated within the transpeptidase domain and clustered around the catalytic region. These residues notably included Thr446 and neighboring residues near positions 412/422/426–427/438/455/480/489, as well as more distal positions such as 597 and 630. As in PBP1a, these candidates were spatially proximal to the catalytic motifs but did not overlap with the conserved catalytic residues themselves.

In PBP2x (Figure 7C), the candidates were densely distributed surrounding the active site region of the transpeptidase domain. Nearly fixed substitutions such as Met385 and Asp567 were retained by the structure-guided filter, as were multiple additional residues surrounding the catalytic cleft (e.g., 382/384/389/401/516/565/576/605). Together, these sites formed a compact motif-proximal cluster framing the catalytic center.

Collectively, the structure-guided screen defined a concise set of high-confidence, noncatalytic, motif-adjacent substitutions in PBP1a, PBP2b, and PBP2x. These residues were recurrent among isolates exhibiting reduced susceptibility to penicillin and were spatially positioned to influence the antibiotic-binding environment without disrupting the conserved catalytic cores. Together, these substitutions define a regional mutational landscape of PBPs and may contribute to the reduced susceptibility to penicillin observed among clinical *S. pneumoniae* isolates from Southwest China.

We further explored the relationship between the PBP variation landscape and isolate-specific PEN-MIC values. The alignment-wide analysis included 144 high-support variable native positions in PBP1a, 96 in PBP2b, and 188 in PBP2x, for a total of 428 variable positions. Multiple site-wise associations with ordered MIC were detected before accounting for population structure; however, no individual position remained significant after GPSC-stratified testing and correction across the complete set of variable sites. A similar pattern was observed at the sequence-profile level. The combined full-sequence profile of PBP1a, PBP2b, and PBP2x was associated with ordered MIC before lineage control (Kruskal–Wallis *H* = 25.93, Holm-adjusted *p* = 0.003), but not after GPSC-stratified permutation (empirical *p* = 0.430; Holm-adjusted *p* = 1.000). Profiles constructed from the 34 structure-guided candidate positions listed in Table 1 yielded the same overall pattern: an unadjusted association with MIC that was not retained after GPSC stratification.

To provide an interpretable summary of recurrent motif-related combinations, we next examined exact profiles formed by the 12 focused PBP positions. Four major profiles, each represented by at least 30 isolates, accounted for 160/204 isolates (78.4%) and included 16/17 isolates with a PEN MIC ≥4 μg/mL (Supplementary Table 4, Supplementary Figure 1). The profile comprising PBP1a T371S/P432T/T574N/S575T/Q576G/F577Y, PBP2b T446A/A619G, and PBP2x T338A/M339F/L546V (KP1; *n* = 35) showed the highest MIC distribution, with a MIC₅₀/MIC₉₀ of 2/4 μg/mL and 8/35 isolates (22.9%) having a MIC ≥4 μg/mL. KP2, which differed from KP1 at the focused PBP2x position 339, comprised 32 isolates and had a MIC₅₀/MIC₉₀ of 2/2 μg/mL, with 3/32 isolates (9.4%) having a MIC ≥4 μg/mL. KP3 (*n* = 49) had a MIC₅₀/MIC₉₀ of 1/2 μg/mL and included 3/49 isolates (6.1%) with a MIC ≥4 μg/mL. KP4, comprising PBP1a T371A/P432T/T574N/S575T/Q576G/F577Y, PBP2b T446A, and PBP2x T338A/L546V (n = 44), had a MIC₅₀/MIC₉₀ of 0.5/2 μg/mL, and 2/44 isolates (4.5%) had a MIC ≥4 μg/mL. The remaining 44 isolates carried heterogeneous, less frequent profiles and had a MIC₅₀/MIC₉₀ of 0.5/2 μg/mL, with 1/44 isolate (2.3%) having a MIC ≥4 μg/mL.

The overall association between the focused exact profiles and ordered MIC was significant before lineage control (Kruskal–Wallis *H* = 29.46, Holm-adjusted *p* < 0.001). However, KP1 and KP2 were both confined to GPSC1, whereas KP3 and KP4 occurred across multiple lineage backgrounds. After MIC values were permuted within GPSCs, the profile-level association was no longer significant (empirical *p* = 0.403; Holm-adjusted *p* = 1.000). Consistent with this result, the total non-TIGR4 residue burden across the three PBPs correlated positively with ordered MIC in the unadjusted analysis (Spearman’s *ρ* = 0.361, Holm-adjusted *p* < 0.001), but the association was not retained after GPSC-stratified permutation (Holm-adjusted *p* = 0.678). No individual site, insertion event, structure-guided candidate profile, or combined PBP profile remained significantly associated with MIC after controlling for GPSC and multiple testing. Thus, the PBP profiles provided descriptive stratification of the penicillin MIC distribution, but the apparent genotype–phenotype associations were strongly influenced by clonal lineage structure.

## 4 Discussion

Penicillin and other β-lactams remain foundational therapies for pneumococcal infections in contemporary practice guidelines, particularly for cases of community-acquired pneumonia where narrow-spectrum β-lactam options (e.g., amoxicillin/penicillin-class agents, depending on the setting and severity) are recommended when clinically appropriate (6, 7). However, the expanding pool of *S. pneumoniae* isolates with elevated penicillin minimum inhibitory concentrations complicates empirical decision-making and is consistent with the broader, well-documented global health burden attributable to bacterial antimicrobial resistance (3).

In this study, we adopted an explicit, disease-agnostic operational definition to ensure internal consistency for downstream genomic analyses: clinical *S. pneumoniae* isolates exhibiting reduced susceptibility to penicillin were defined as isolates with penicillin minimum inhibitory concentrations ≥0.12 μg/mL, with the CLSI meningitis threshold used as a clinically motivated screening/enrichment criterion (31, 32). This choice reflects the observations that the CLSI penicillin interpretive criteria are intentionally different for meningitis and nonmeningitis syndromes and have undergone iterative updates and that clinical management often requires early escalation decisions before definitive confirmation of central nervous system infection (e.g., cerebrospinal fluid culture) is available (33, 34). Given that pneumococcal infections can progress from invasive infections and bacteremia to meningitis, applying a unified MIC-based inclusion threshold allows the evaluation of a biologically coherent cohort enriched for isolates with elevated penicillin MICs without the internal inconsistency that would arise from the simultaneous application of two markedly different syndrome-specific breakpoint frameworks to define “susceptibility” within the same analysis set (35, 36).

Among the 204 clinical *S. pneumoniae* isolates enriched for elevated penicillin MICs (PEN MIC ≥0.12 μg/mL) in our cohort, the most notable coresistance pattern was near-universal macrolide resistance (erythromycin, 98.5%) together with very high tetracycline resistance (82.8%) and substantial co-trimoxazole resistance (64.7%), whereas fluoroquinolone activity remained largely preserved, and no vancomycin/linezolid resistance was detected. This “elevated β-lactam MIC plus multidrug coresistance” profile aligns with contemporary surveillance showing that resistance burdens in pneumococci often cluster within successful genetic backgrounds, thereby limiting the practical oral step-down options after β-lactam susceptibility is compromised. In recent U.S. multicenter surveillance datasets that were not selected by the penicillin MIC, the resistance rates were substantially lower than those in our penicillin MIC-enriched cohort—for example, the macrolide resistance rate was ~37.7%, the penicillin nonsusceptibility rate was ~22.1%, and the tetracycline resistance rate was ~16.1% in a large multicenter analysis, with similarly moderate rates also reflected in ABC-derived summaries (e.g., an erythromycin nonsusceptibility, ~29.3%; TMP–SMX nonsusceptibility, ~18.2%; and tetracycline nonsusceptibility, ~12.5%), highlighting how MIC-based enrichment concentrates MDR phenotypes when the analytic focus is elevated penicillin MICs (37). In contrast, a multicenter hospital-based study of Chinese adults reported resistance rates exceeding 90% for macrolides, 89.6% for tetracycline, and 58.1% for TMP–SMX, indicating a similarly high burden of non-β-lactam resistance in a contemporary Chinese pneumococcal population (38). Contemporary clinical series in European adults also show persistent macrolide resistance but generally lower TMP–SMX resistance rates (e.g., ~40.5%, ~13.8%, and ~12.1% for erythromycin, TMP–SMX, and penicillin, respectively), while vancomycin and linezolid activity is universally retained—again, underscoring the idea that our MIC-enriched cohort represents a clinically relevant high-resistance subset rather than background pneumococcal populations (39).

By leveraging the PubMLST Pneumococcal Genome Library, we were able to contextualize our regional collection against a large, internationally sampled genome resource processed through the same QC and annotation pipeline while holding the inclusion criterion constant. Because the pneumococcal capsular antigenic composition is strongly structured by the phylogenetic background and can be shaped by local vaccine uptake and antibiotic selection, such database-anchored comparisons provide a practical framework for quantifying between-country heterogeneity within a clinically enriched subset rather than inferring population-level prevalence from area series (40, 41).

Within this penicillin MIC-enriched analytic framework, the serotype composition differed markedly across Southwest China, the PubMLST-USA dataset, and the PubMLST-Thailand dataset. These patterns were consistent with the broader post-PCV era concept that vaccine pressure can reduce the prevalence of disease caused by vaccine types while creating ecological space for the expansion of nonvaccine serotypes and lineages, some of which may preferentially concentrate resistance determinants, yet the direction and magnitude of replacement are region-specific and depend on local program implementation, uptake, and circulating clonal backgrounds (42, 43). In this context, it is noteworthy that pneumococcal vaccine policies and program maturity differ substantially across the three regions: the United States has long implemented routine childhood PCV immunization and, more recently, has expanded the adult recommendations to higher-valency PCVs in the updated ACIP guidelines (44); in contrast, in China, PCV13 remains outside the National Immunization Program in most settings and is largely administered as a self-paid (non-NIP) vaccine (45), and a report indicated that Thailand had not incorporated pneumococcal vaccines into the National Immunization Program as of 2022 despite guideline-level recommendations and documented but limited uptake (46). These policy differences provide epidemiological context but should not be interpreted as direct causal explanations for the observed serotype differences, because the sampling periods and source populations were not harmonized across countries. Collectively, these comparisons highlight that, even under a harmonized MIC-based definition, the serotype composition of isolates with elevated penicillin MICs is highly geographically dependent; therefore, regional clinical genomic datasets benefit from explicit international contextualization using standardized resources while maintaining careful interpretation boundaries with respect to representativeness and prevalence.

The incorporation of Global Pneumococcal Sequence Clusters (GPSCs) provides an internationally comparable genomic framework that complements serotyping by capturing deeper lineage relatedness, thereby helping to interpret geographic heterogeneity, vaccine impact, and lineage-associated antimicrobial resistance at a scale that is less sensitive to serotype switching than capsule-based classification alone is (20). In our Southwest China cohort of clinical *S. pneumoniae* isolates exhibiting reduced susceptibility to penicillin, we observed a concentrated lineage composition dominated by GPSC1 and including several secondary lineages (e.g., GPSC4, GPSC23, and GPSC69). Importantly, the nested correspondence between MLST types and GPSCs in our dataset supports the internal coherence of the lineage assignments and indicates that, within this regional population with reduced penicillin susceptibility, the major STs largely represent expansions of a limited number of successful GPSC backgrounds.

When contextualized against the filtered PubMLST genomes, the GPSC spectrum differed markedly among Southwest China, the United States, and Thailand, with a very large effect size (Cramér’s *V*). Such strong between-country structuring is consistent with large-scale genomic surveillance data demonstrating that pneumococcal populations are typically composed of a limited set of globally distributed lineages whose local dominance varies by geography, antibiotic selection pressure, and vaccine history (20, 42). Notably, while the United States dataset was enriched for lineages such as GPSC5, GPSC59, and GPSC904;9, the Southwest China and Thailand datasets shared the more limited feature that GPSC1 was the most common lineage in both settings. This partial concordance suggests that certain lineages may have increased fitness in specific regional ecologies (e.g., antibiotic use patterns and transmission networks) but also highlights the concept that lineage replacement can be setting-specific and is not fully predicted by serotype frequencies alone (42). Because PubMLST includes heterogeneous sampling designs across countries, we interpret these contrasts as evidence of geographic lineage structuring within the analyzed elevated-MIC subsets, while acknowledging that the magnitude of the observed differences may be influenced by surveillance intensity and case mix.

The combined core genome phylogeny further reinforces the utility of GPSC-based interpretation. Across the global-plus-local tree, GPSCs formed well-demarcated clades, and serotypes were aggregated nonrandomly along major lineages, indicating that capsule types are constrained by the genetic background at the clade scale even though capsular switching can occur (20, 42). In our dataset, several dominant serotype–GPSC associations were evident (e.g., 19F/19A with GPSC1; 23A/23B with GPSC5; and 6A/6B with GPSC23), emphasizing that regional serotype distributions are, to a substantial degree, a reflection of the lineages that are currently expanding. This idea matters for inference: shifts in serotype prevalence may represent the expansion of preexisting lineages carrying particular capsules, true capsular switching within persistent lineages, or both—each with different implications for predicting antimicrobial resistance trajectories and the durability of vaccine impact (42).

Although the penicillin MIC categories were intermingled across the phylogeny, the lineage-level tendencies we observed—such as the relative scarcity of very high MICs in GPSC5 and GPSC904;9 compared with the broader MIC spectrum in GPSC16—support a model in which β-lactam susceptibility is shaped by the lineage background and its evolutionary “solution space” (e.g., recombination and mosaic events in PBPs) rather than being strictly determined by serotype alone. The weaker, overlapping association between serotype and MIC in our analysis is therefore not unexpected and further supports the use of GPSCs as an analytic layer to interpret resistance phenotypes beyond capsule typing (20, 42).

In contrast to the GPSC distribution and phylogenetic context, our virulence gene analysis was intentionally restricted to the 204 locally collected clinical isolates. A key limitation of PubMLST for this purpose is that for many entries, the clinical metadata are insufficient to reliably distinguish invasive/pathogenic isolates from carriage or nondisease isolates; without robust disease attribution, the interpretation of “virulence enrichment” risks conflating true pathogenicity determinants with lineage markers of colonization ecology. By focusing on clinically sourced isolates with harmonized phenotyping and a consistent collection context, we aimed to ensure that associations between the GPSC background and virulence/resistance profiles are interpreted within a clinically relevant framework.

Within these clinical isolates, we observed a clear dichotomy between (i) near-universal core virulence loci and (ii) highly variable colonization-associated loci whose presence/absence was strongly structured by GPSC. The striking enrichment of both the pilus-1 and pilus-2 operons within GPSC1 is particularly notable because pneumococcal pili have been experimentally linked to increased adhesion, inflammation, and virulence in animal models and are recognized as lineage-associated accessory traits that can contribute to clonal success (47, 48). In this context, the near-fixation of pilus-2 and the high prevalence of pilus-1 within GPSC1 in our cohort may represent a lineage-level fitness package that facilitates transmission and persistence under local selective pressures, thereby indirectly supporting the high observed frequency of GPSC1 isolates among the clinical isolates with reduced penicillin susceptibility.

The resistance gene patterns further support the occurrence of lineage-structured selection rather than random acquisition. The near-universal presence of macrolide and tetracycline determinants is consistent with sustained antimicrobial pressure, but the GPSC-stratified contrasts—especially the strong enrichment of *mefA/msrD* and folate-pathway determinants (*folA/folP*) within GPSC1—suggest that a subset of lineages may carry stable mobile genetic elements and/or background-specific resistance architectures that are maintained during clonal expansion. This interpretation aligns with prior work in CC271-associated pneumococci showing that dual macrolide resistance determinants can be colocalized on composite elements (e.g., Tn2010 carrying *ermB* and *mefE*), facilitating coselection and rapid dissemination under macrolide exposure (49). More broadly, reviews of pneumococcal macrolide resistance have emphasized that the local dominance of particular lineages can strongly shape the prevalence of population-level resistance because efflux- and methylase-mediated mechanisms are often embedded within successful clonal backgrounds (50).

Reduced penicillin susceptibility in pneumococci is ultimately mediated by altered β-lactam targets; thus, PBPs provide the most mechanistically direct explanation for the MIC elevation patterns observed across GPSCs. Contemporary syntheses have emphasized that pneumococcal β-lactam resistance is rarely driven by single breakpoint mutations; instead, it typically reflects stepwise remodeling of multiple cell wall loci—most prominently, mosaic *pbp1a/pbp2b/pbp2x* alleles generated by recombination with mitis group streptococci—often accompanied by compensatory changes in additional peptidoglycan biogenesis determinants (e.g., *murM* and related pathways) that stabilize fitness during clonal expansion under β-lactam exposure (51–53). In this context, our site-resolved analysis of *pbp1a*, *pbp2b*, and *pbp2x* across the 204 clinical isolates enriched for a PEN MIC ≥0.12 μg/mL provides a region-specific mutational landscape.

From an epidemiological perspective, two features of the variation profiles are informative when these profiles are compared with patterns reported in other settings. First, substitutions were not evenly distributed but were instead clustered within the C-terminal transpeptidase domains and surrounding the conserved catalytic motifs [SXXK, SXN, and K(T/S)G], a pattern consistent with global observations that β-lactam resistance evolves through motif-proximal remodeling while essential catalytic residues are preserved (51, 53, 54). Second, several nearly fixed hotspots in our Southwest China cohort were located at positions within widely recognized signature regions used in genomic prediction frameworks and PBP typing approaches. Specifically, the high-frequency changes at PBP1a T371 and within the TSQF block (T574N, S575T, Q576G, and F577Y), PBP2b T446, and PBP2x T338 overlapped previously described β-lactam resistance-associated signature positions or regions (54, 55). This concordance provides literature-based context for our site-specific profiles, although the observed frequencies in this MIC-enriched cohort do not by themselves establish an independent causal contribution of each substitution to reduced β-lactam susceptibility. Together, these findings support the inference that the Southwest China collection of isolates exhibiting reduced susceptibility to penicillin shares core evolutionary solutions with globally circulating pneumococci exhibiting reduced β-lactam susceptibility, while the specific constellation and fixation level of the sites observed herein likely reflects the local lineage structure (notably, GPSC1 dominance) and region-specific antimicrobial selection.

The exploratory genotype–phenotype analysis further showed that the relationship between PBP variation and penicillin MIC cannot be interpreted independently of the clonal background. Although the combined PBP profiles and residue burden stratified the MIC distribution before lineage control, the higher-MIC profiles were concentrated within GPSC1, and the associations were not retained after GPSC-stratified permutation. Moreover, isolates sharing the same focused PBP profile still spanned multiple MIC dilutions. These findings do not exclude a mechanistic contribution of coordinated PBP remodeling but indicate that the present observational dataset cannot assign a specific MIC increase to an individual substitution or fixed combination. Linked variation elsewhere within the mosaic PBP sequences, other cell-wall-related loci, and lineage-specific genomic backgrounds may jointly modify the phenotype.

Mechanistically, the detailed topology of our candidate sites provides a structurally plausible hypothesis in which reduced susceptibility may involve geometric and electrostatic remodeling of the active-site environment rather than disruption of the catalytic machinery. In all three PBPs, the conserved catalytic residues themselves remained intact, whereas high-frequency substitutions accumulated on surface-exposed loops and helices that frame the active site cleft. Such motif-adjacent changes could potentially influence β-lactam acylation kinetics by altering (i) the accessibility of the binding groove, (ii) the hydrogen-bonding networks that stabilize β-lactam positioning, and (iii) the local conformational dynamics that govern the transition state of the transpeptidase reaction—thereby lowering drug affinity or reactivity while preserving the endogenous transpeptidase function required for viability (51, 53). The particularly strong diversification at the second residue of the SXXK motif in PBP1a and PBP2x is notable in this regard: these substitutions lie immediately adjacent to the catalytic serine and could plausibly affect local backbone conformation and side-chain packing around the nucleophilic residue without replacing the serine itself (51, 53). These structural interpretations are hypothesis-generating and do not establish an independent biochemical effect for any individual substitution.

Our structure-guided filtering further refined this mechanistic interpretation by prioritizing noncatalytic residues that are simultaneously (i) among the most variable positions and (ii) located within a short Cα distance of a catalytic motif. The resulting compact sets of candidate residues in PBP1a, PBP2b, and PBP2x formed motif-proximal shells around the active site, identifying positions at which substitutions could plausibly influence the local β-lactam-binding environment. The differing architecture of variability across the PBPs was also compatible with a multilocus, coordinated resistance model: PBP2b contains relatively conserved catalytic motifs with hotspots concentrated in the surrounding regions, whereas PBP1a and PBP2x show more pronounced motif-adjacent diversification, which is consistent with the idea that clinically relevant MIC increases result from complementary remodeling across multiple PBPs rather than from a single dominant target alteration (51–54). However, recurrence and structural proximity alone do not demonstrate that these sites independently increase the MIC or that a particular combination is sufficient to produce the phenotype. Such coordinated remodeling is also biologically consistent with experimental and evolutionary evidence indicating that high-level β-lactam resistance can require the ordered acquisition of multiple loci through transformation, followed by selection and stabilization within expanding clonal backgrounds (52, 53).

Finally, our Southwest China-specific site map has practical implications for surveillance and translation. Recent work has underscored the feasibility of predicting β-lactam susceptibility directly from genome sequences by leveraging mosaic patterns in PBPs, offering a path toward rapid, standardized genomic AST in pneumococci. By providing a concise, high-confidence catalog of motif-adjacent, recurrent substitutions in a clinically enriched regional cohort, anchored to TIGR4 numbering and supported by structural proximity, our study supplies empirically grounded candidates for region-tailored monitoring and for future functional validation. Our GPSC-stratified analyses further showed that the apparent associations of combined PBP profiles and residue-substitution burden with penicillin MIC were no longer significant after lineage control. These findings illustrate the importance of integrating candidate-site information with GPSC context when attempting to distinguish substitutions that track successful resistant lineages from variants that may independently influence MIC, particularly when genomic prediction tools are applied across settings with different lineage compositions.

This study adds value in three pragmatic ways: first, it applies a harmonized, disease-agnostic MIC-based enrichment definition and a single QC/annotation pipeline to enable apples-to-apples international comparisons within an elevated-MIC subset; second, it integrates the serotype, GPSC, and phylogeny so that geographic heterogeneity is interpreted through a stable lineage structure rather than through capsule typing alone; and third—and most importantly—to our knowledge, it is the first to delineate a Southwest China-specific, site-resolved PBP variation landscape among clinical *S. pneumoniae* isolates exhibiting reduced susceptibility to penicillin. By quantifying residue-level variability in *pbp1a/pbp2b/pbp2x* under a unified TIGR4 numbering scheme and applying a structure-guided prioritization approach focused on motif-proximal, noncatalytic positions, we provide a concise set of recurrent candidate substitutions that frames the current regional state of PBP remodeling and offers a practical starting point for surveillance and functional follow-up.

Several caveats regarding interpretation must be considered: because the cohort was intentionally MIC-enriched, the reported serotype/GPSC and resistance patterns should be viewed as properties of a clinically relevant reduced susceptibility subset rather than the population prevalence; and the PubMLST-based contrasts among the countries may be influenced by heterogeneous sampling and limited clinical metadata. Virulence-gene carriage was inferred from sequence homology using a predefined identity threshold; therefore, detection indicated the presence of a homologous sequence but did not establish that the gene was intact or functionally expressed. Potentially disruptive SNPs, insertions, deletions, frameshifts, premature stop codons, and regulatory variants were not systematically evaluated, and transcriptional, protein-level, or phenotypic validation was not performed. Accordingly, the virulence profiles reported here should be interpreted as predicted gene-detection patterns rather than direct evidence of functional virulence-factor expression. In addition, because all 204 isolates were selected using a PEN MIC threshold of ≥0.12 μg/mL, the cohort lacked a fully susceptible comparison group with MICs ≤0.06 μg/mL, limiting estimation of the independent effects of substitutions that were already nearly fixed in this MIC-enriched collection. The PBP genotype–phenotype analysis was restricted to penicillin MIC and should not be extrapolated quantitatively to other β-lactam agents. Strong collinearity between PBP profiles and GPSCs, together with the absence of functional validation, also precluded causal attribution of MIC elevation to a specific substitution or PBP combination.

## 5 Conclusion

In this study, we characterized 204 clinical *S. pneumoniae* isolates with reduced susceptibility to penicillin (PEN MIC ≥0.12 μg/mL) collected from five institutions in Southwest China using whole-genome sequencing. Within this clinically defined reduced susceptibility cohort, macrolide resistance was nearly universal and accompanied by high tetracycline and co-trimoxazole resistance, while the activity of fluoroquinolones, vancomycin, and linezolid was largely retained. Molecular serotyping revealed the predominance of vaccine-associated serotypes, particularly 19F and 19A, with estimated PCV13 and PPV23 serotype coverage of 69.6 and 65.2%, respectively. Lineage analysis further indicated that GPSC1 was the most common genetic background, and harmonized comparisons within a PubMLST-derived elevated-MIC subset highlighted the substantial geographic heterogeneity in lineage composition across countries. The core genome phylogeny and accessory genome annotation supported the strong lineage structuring of colonization-associated virulence loci and resistance determinants, including marked enrichment of pilus operons and *mefA/folA/folP* within GPSC1.

At the penicillin-binding protein level, the site-specific variability in *pbp1a*, *pbp2b*, and *pbp2x* was concentrated in the transpeptidase domains and near conserved catalytic motifs, while the essential catalytic residues themselves remained conserved. A compact set of highly variable, motif-proximal noncatalytic residues across the three PBPs was prioritized via a structure-guided screen. Although combined PBP profiles descriptively stratified the penicillin MIC distribution, these associations were not retained after GPSC-stratified analysis, indicating strong lineage confounding and precluding attribution of a specific MIC increase to an individual substitution or fixed PBP combination. Together, these findings provide a regional catalog of candidate PBP sites that can support targeted surveillance and subsequent functional validation.

## Data Availability

The datasets presented in this study can be found in online repositories. The names of the repository/repositories and accession number(s) can be found below: https://www.ncbi.nlm.nih.gov/genbank/, PRJNA914101, PRJNA915833, PRJNA915821.

## Footnotes

1
https://pathogen.watch

## Supplementary material

SUPPLEMENTARY FIGURE 1 Penicillin MIC distributions across combined PBP key-site profiles. Stacked horizontal bars show the within-profile distributions of PEN-MIC values among the 204 clinical Streptococcus pneumoniae isolates exhibiting reduced susceptibility to penicillin collected in Southwest China. Combined profiles were defined by the exact amino acids at 12 focused positions, reported using native ungapped TIGR4 numbering: PBP1a positions 371, 432, and 574–577; PBP2b positions 446 and 619; and PBP2x positions 338, 339, 394, and 546. KP1–KP4 represent the four exact profiles detected in at least 30 isolates; less frequent profiles were pooled as “Other profiles” for descriptive presentation. Each colored segment indicates the percentage of isolates within that profile assigned to the corresponding ordered twofold-dilution MIC category, and the total number of isolates in each group is shown on the right. The ≥8 μg/mL category represents the highest censored MIC category.
